# Data set of a representative online survey on search engines with a focus on search engine optimization (SEO): a cross-sectional study

**DOI:** 10.12688/f1000research.109662.2

**Published:** 2022-09-12

**Authors:** Sebastian Schultheiß, Dirk Lewandowski

**Affiliations:** 1 Department of Information, Hamburg University of Applied Sciences, Hamburg, 22081, Germany

**Keywords:** Search engines, search engine optimization (SEO), paid search marketing (PSM), online survey, user studies, searcher attitudes, awareness, external influences

## Abstract

To gain a better understanding of user knowledge and perspectives of search engines, a fruitful approach are representative online surveys. In 2020, we conducted an online survey with a sample representative of the German online population aged 16 through 69 ( *N* = 2,012). The online survey included 12 search engine-related sections. The questions cover topics such as usage behavior, self-assessed search engine literacy, trust in search engines, knowledge of ads and search engine optimization (SEO), ability to distinguish ads from organic results, assessments and opinions regarding SEO, and personalization of search results. SEO is the specific focus of the survey, as it was conducted as part of the SEO Effect project, dealing with issues such as the role of SEO from the user perspective. This data set contains complete data from the online survey. On the one hand, the data set will allow further analyses, and, on the other hand, comparisons with follow-up studies.

## Introduction

Representative surveys are suitable for gaining a better understanding of how users interact with search engines, how they understand them, and what opinions they have about them. However, such studies are quite rare and usually refer to individual subareas, such as frequency of use ( Beisch & Schäfer, 2020) or trust in search engines ( Edelman, 2020), while ignoring other areas, such as paid-search marketing (PSM) and search engine optimization (SEO).

SEO “is the practice of optimizing web pages in a way that improves their ranking in the organic search results” ( Li *et al*., 2014). The SEO industry is one of the major stakeholder groups regarding search results of commercial search engines like Google ( Röhle, 2010). Although the SEO industry generates billions in revenue ( tbrc.info, 2021), little is known about whether search engine users are aware of SEO and what they think about it.

To close this gap, we conducted an online survey in 2020 with a sample representative of the German online population. Questions on SEO are the focus of the survey, as it was conducted as part of the SEO Effect project, funded by the German Research Foundation. The overall goal of the project is to describe and explain the role of SEO from the perspective of the participating stakeholder groups, one of them being the users. A total of 999 people participated in the online survey on a large screen (e.g., desktop PC), and 1,013 on a small screen (smartphone). The online survey included several search engine-related sections ( Schultheiß *et al*., 2022). Some of the questions were self-developed and others were adopted from other studies. This data set contains the full data from the online survey.

## Materials and methods

We conducted a representative online survey with German internet users. The survey was carried out as part of the SEO Effect project in cooperation with the market research company Fittkau & Maaß Consulting (hereinafter abbreviated as F&M) between March and April 2020. F&M performed the following services, all in consultation with the project team:

• programming of the survey using FileMaker as a database (January 13 - February 27, 2020)
• conducting of the survey (March 2 – April 9, 2020)
• data analysis and reporting (April 2020)

The subjects were recruited through the online panel provider respondi, which is in cooperation with F&M. An online panel is a sample database with a large number of people (often one million or more). These people have agreed to be available as potential respondents in surveys, as long as they meet the selection criteria for the particular study ( Callegaro *et al*., 2014). In the next section, the sample is discussed in detail.

### Sampling

We used a sample that is representative of the German online population according to the criteria applied by “Arbeitsgemeinschaft Onlineforschung” ( working group online research; AGOF). For sampling, the characteristics age, gender, and state were used. The population includes German internet users from the age of 16 to 69 years. Based on two subsamples to be formed (see below), both of which had to meet the same requirements regarding representativeness, we intended a minimum sample size of *N* = 2,000 subjects (recommended by F&M) and achieved a sample size of *N* = 2,012 subjects.

From the total sample, two sub-samples of *N* = 999 subjects (large screen) and *N* = 1,013 subjects (small screen) were formed, which meet the same requirements regarding representativeness described above. Sample 1 attended the survey with a large screen (e.g., desktop PC, laptop, tablet; group “large screen”), sample 2 with a smartphone (group “small screen”).

To assign the subjects to one of the two groups, the panel provider detected the user agent string to determine which device and browser the potential subject was using and assigned the participants accordingly. The correct assignment of the test persons was checked by respondi and F&M. The online panel provider respondi checked the devices used by the subjects before forwarding them to the questionnaire. In addition, the devices used by the subjects were verified by F&M as part of the plausibility check of the data by using the user agent string. The subjects were invited to the survey by e-mail. Each participant received 0.75 euro for complete participation. Since we used a sample that is representative of the German online population, we do not assume biases regarding the composition of the sample. However, it should be mentioned that the online survey may have also addressed people who participated solely because of the compensation.

### Questionnaire

First, we developed a catalogue of questions. We derived questions for the survey from the objectives of the “SEO Effect” project, from findings of expert interviews ( Schultheiß & Lewandowski, 2021d), and from literature research (In Scopus, we searched for surveys that included “search engine” and “information literacy” (or synonyms)). After preparing the questions, we sent them to the market research company (F&M). F&M made recommendations regarding the sequence and formulation of the questions as well as suggestions for new questions, which we included.

In several feedback rounds, we jointly created the final version of the questionnaire (see Table 1). In the introduction to the survey, we first welcomed the respondent and thanked him/her for participating. We also pointed out that the questionnaire is used exclusively for research purposes and that by participating, the respondent agrees to the attached privacy policy of F&M.

**Table 1.** Questionnaire.

Section	No.	Question	Response options of original study	Response options final (adapted/translated if necessary)	Comments	Ref.
I) Screening	1.1	How old are you?	/	- under 16 years - 16 to 19 years - 20 to 24 years - 25 to 29 years - 30 to 34 years - 35 to 39 years - 40 to 44 years - 45 to 49 years - 50 to 54 years - 55 to 59 years - 60 to 64 years - 65 to 69 years - 70 years and older	For quotation purposes; exclusion of subject if under 16 years of age or 70 years and older.	9
	1.2	You are …	/	- Female - Male	For quotation purposes	9
	1.3	Which state do you live in?	/	- Baden-Württemberg - Bayern - Berlin - Brandenburg - Bremen - Hamburg - Hessen - Mecklenburg-Vorpommern - Niedersachsen - Nordrhein-Westfalen - Rheinland-Pfalz - Saarland - Sachsen - Sachsen-Anhalt - Schleswig-Holstein - Thüringen	For quotation purposes	9
II) Usage behavior	2.1	What do you use the internet for?	/	Please mark all applicable answers: - Browsing the web, e.g., for entertainment, to pass the time - Search for something - Read news, reports, articles - Use social networks, communities, e.g., Instagram, Facebook - Communicate via e-mail, messenger - Online shopping/ordering/booking - Online banking/broking - Watch movies, videos - Listen to and download music - Gaming - Other		9
	2.2	If you are searching for something online: Which search engine(s) do you usually use?		Please mark all applicable answers: - Bing - Ecosia - DuckDuckGo - Google - Web.de - Yahoo! - Others, namely … (free input) - None	Exclusion of respondent if no search engines are used	8, adjust-ments by: 9
	2.3	Which search engine do you use most often?	- Google - Yahoo Search - Bing - AOL - Ask - Lycos - MyWebSearch - Dogpile - WebCrawler - Other (SPECIFY) - None/Don’t use any regularly - Don’t know - Refused	- Bing - Ecosia - DuckDuckGo - Google - Web.de - Yahoo! - Another - I don’t know/not specified	Only used search engines (according to previous question) are displayed. Question omitted if only one search engine is used.	6, adjustments by: 9
	2.4	Which devices do you use search engines with?	Multiple Choice: - Desktop PC/Laptop - Smartphone - Tablet	Please mark the appropriate answer in each case: - via desktop computer, PC - via laptop - via tablet - via smartphone - via smart speaker (e.g., Amazon Echo, Alexa, Google Home) - *frequently* - *occasionally* - *rarely* - *never* - *I don’t know*		8, adjustments by: 9
	2.5	Why is [search engine] the search engine you use most often? Please mark up to 5 answers.	I use [search engine] most because … - it is easy to use - it is fast - the results list is clearly arranged - it seems objective to me - the most important results are always at the top of the results list - I always find what I’m looking for - I’ve always used it - I think it covers most of the internet - it provides helpful information on the individual results - it does not show any dubious results - I know exactly how it works - it sometimes shows surprising results - it offers the possibility to fade out dubious results - my friends and colleagues also use it - I like the layout and colors of the search engine page - *Scale from 1= not applicable to 4= fully applicable*	I use [search engine] most because … - the results list is clearly arranged - I like the layout and colors of the search engine result page - it is easy to use - it is fast - it seems objective to me - I always find what I’m looking for - I know exactly how it works - I think it covers most of the internet - the most important results are always at the top of the results list - it provides helpful information on the individual results - it sometimes shows surprising results - it does not show dubious hits or these can be hidden - I’ve always used it - my friends and colleagues also use it - I do not know any other search engines - it is the default setting in the browser - no particular reason - Other reason, namely… (free input)	The name of the most frequently used search engine is shown	5, adjustments by: 9
	2.6	Can you estimate how many queries you submit to search engines in a regular week?	- several times a day - about once a day - 3 to 5 days a week - 1 to 2 days a week - once every few weeks - less often - never - don’t know - refused	- more than 100 per week - over 50 to 100 per week - over 20 to 50 per week - over 10 to 20 per week - 6 to 10 per week - 1 to 5 per week - less than 1 per week - I don’t know		6, adjustments by: 9
III) Self-assessment	3.1	When it comes to finding something on the internet using search engines: How do you assess your own abilities in this respect?	- German school grades (1-6)	My skills in search engine usage are… - perfect - excellent - good - fair - bad - I don’t know	Check for correlation between self-assessment and actual knowledge	3, adjustments by: 9
	3.2	And how often do you think you find what you are looking for using search engines?	- always - most of the time - only some of the time - hardly ever - don’t know - refused	I find what I’m looking for… - always - most of the time - sometimes - rarely - never - I don’t know		6, adjustments by: 9
IV) Trust	4.1	If you think of search engines in general: To what extent do you think the following statements apply to search engines?	a) “In general, do you think internet search engines are a fair and unbiased source of information, or do you think search engines are NOT a fair and unbiased source?”: - Yes, they are a fair and unbiased source of information - No, they are NOT a fair and unbiased source of information - Depends - Don’t know - Refused b) “In general, how much of the information you find using search engines do you think is accurate or trustworthy? Would you say…”: - All or almost all - Most - Some - Very little - None at all - Don’t know - Refused	Please mark the appropriate answer in each case: - Search engines are fair and unbiased sources of information - The information I find through search engines is correct and trustworthy - *absolutely correct* - *correct* - *neutral* - *rather not true* - *doesn’t apply at all* - *I don’t know*		6, major adjustments regarding the question structure and responses by: 9
	4.2	And if you think especially of Google: To what extent do you think the following statements apply to Google?	a) “In general, do you think internet search engines are a fair and unbiased source of information, or do you think search engines are NOT a fair and unbiased source?”: - Yes, they are a fair and unbiased source of information - No, they are NOT a fair and unbiased source of information - Depends - Don’t know - Refused b) “In general, how much of the information you find using search engines do you think is accurate or trustworthy? Would you say…”: - All or almost all - Most - Some - Very little - None at all - Don’t know - Refused	Please mark the appropriate answer in each case: - Google is a fair and unbiased source of information - The information I find through Google is correct and trustworthy - *absolutely correct* - *correct* - *neutral* - *rather not true* - *doesn’t apply at all* - *I don’t know*		6, major adjustments regarding the question structure and responses by: 9
V) Query match	5.1	If you think of search engines in general: To what extent do you think the following statement applies to search engines?		- The results displayed in search engines match my queries perfectly - *absolutely correct* - *correct* - *neutral* - *rather not true* - *doesn’t apply at all* - *I don’t know*	Questions 5.1 and 5.2 follow on from the previous questions on trust and were added to the questionnaire in consultation with F&M.	9
	5.2	To what extent do you think the following statement applies to Google?		- The results displayed in Google match my queries perfectly - *absolutely correct* - *correct* - *neutral* - *rather not true* - *doesn’t apply at all* - *I don’t know*		9
VI) Knowledge of search result influences	6.1	When it comes to the search results displayed on Google: What do you think influences the ranking of search results on Google?		- The Google search results and their ranking depend on… (free input) - I don’t know		9
VII) Knowledge of ads	7.1	What do you think: Where does Google generate most of its revenue from?		- Google generates revenue primarily through… (free input) - I don’t know		3
	7.2	Do website operators or companies have the opportunity to pay for their results to appear high up on Google’s search results page?		- Yes, this is possible - No, that possibility does not exist - I don’t know		3
	7.3	Do such paid search results differ from the other search results?		- Yes, you can recognize them and distinguish them from the other search results - No, they cannot be identified - I don’t know	[If “Yes” on previous question]	3
	7.4	And how do the paid search results on Google differ from the other results that have not been paid for?		- The paid search results on Google can be recognized by… (free input) - I don’t know	[If “Yes” on previous question]	3
VIII) Knowledge of SEO	8.1	Do website operators or companies have the ability or influence to appear higher in the Google results list for certain queries without paying any money to Google?		- Yes, this is possible - No, that possibility does not exist - I don’t know		1
	8.2	Do you know what term is used to describe these measures to improve the ranking in the Google search results list (without payment to Google)?		- Yes, this is called… (free input) - I don’t know	[If “Yes” on previous question]	1
	8.3	And by what means can a website be designed or programmed so that it is ranked higher in the Google search results lists?		Please enter all possibilities/measures that you know: - With the help of the following measures: … (free input) - I don’t know	[If “Yes” on question 8.1] Serves for further differentiation of SEO knowledge levels	1
		Information part “SEO/PSM“: Website operators have several ways to ensure that their web pages appear at the top of the Google result page for a specific query, namely I) Payment: They pay money to Google*, or II) Search engine optimization: They design their websites accordingly, e.g., by using certain keywords, quick page speed, and appropriate image titles and descriptions. Next, we will show you two different Google result pages and would like to ask you whether or which results can be influenced by payment to Google and/or search engine optimization.				10, adjustments by: 9
IX) Ability to distinguish ads from organic results	9.1	You will now see a Google results page. Are there any search results on this page that can be influenced by the website operator paying Google?		- No, there are no search results on this page that can be influenced by payments to Google - Yes, the following search results can be influenced by paying money to Google: *Please click on the corresponding search results*	SERP screenshot from **block I** (A or B) to mark all **ads**	3
	9.2	One more question about this search results page: Are there any search results on this page that can be influenced by search engine optimization?		- No, there are no search results on this site that can be influenced by search engine optimization - Yes, the following search results can be influenced by search engine optimization: *Please click on the corresponding search results*	SERP screenshot from **block I** (A or B) to mark all **organic results**	1
	9.3	You will now see another Google results page. Are there any search results on this page that can be influenced by the website operator paying Google?		- No, there are no search results on this page that can be influenced by payments to Google - Yes, the following search results can be influenced by paying money to Google: *Please click on the corresponding search results*	SERP screenshot from **block II** (C or D) to mark all **ads**	3
	9.4	One more question about this search results page: Are there any search results on this page that can be influenced by search engine optimization?		- No, there are no search results on this site that can be influenced by search engine optimization - Yes, the following search results can be influenced by search engine optimization: *Please click on the corresponding search results*	SERP screenshot from **block II** (C or D) to mark all **organic results**	1
X) Assessments and opinions regarding SEO	10.1	Now please think again about search engine optimization. In your opinion, how strong is the influence of search engine optimization on the ranking of the search results in Google?		Influence of search engine optimization on the order of search results in Google: - *very strong influence* - *major influence* - *medium influence* - *little influence* - *no influence* - *I don’t know*		1
	10.2	How big are the positive and negative effects of search engine optimization on the Google search results from your perspective?		Please mark the appropriate answer in each case: - I perceive the positive effects of search engine optimization as … - I perceive the negative effects of search engine optimization as … - *very large* - *large* - *medium* - *low* - *non-existent* - *I don’t know*		1
	10.3	Which positive effects does search engine optimization have in your opinion?		- I assess the following effects of search engine optimization as positive: … (free input) - I can’t say	Question to internet users who see high or very high positive SEO effects	9
	10.4	Which negative effects does search engine optimization have in your opinion?		- I assess the following effects of search engine optimization as negative: … (free input) - I can’t say	Question to internet users who see high or very high negative SEO effects	9
XI) Personalization	11.1	If a search engine records your search queries and uses this information to customize search results for you in the future: What do you think about that?	- It’s a bad thing if a search engine collected information about your searches and then used it to rank your future search results, A: because it may limit the information you get online and what search results you see B: because you feel it is an invasion of privacy - It’s a good thing if a search engine collected information about your searches and then used it to rank your future search results, A: because it gives you results that are more relevant to you B: even if it means they are gathering information about you - Neither of these - Don’t know - Refused	- I think that's a positive thing - neutral - I think that's a negative thing - I don’t know/not specified		6, adjustments by: 9
	11.2	And have you ever taken measures to limit the amount of data that search engines collect about you? If so, which ones?	- Changed your browser settings - Deleted your web history - Used the privacy settings of websites *- Yes* *- No* *- Don’t know* *- Refused*	Please mark all applicable answers: - Deleted past activities (for example, search history) - Disabled storage of future activities (e.g., search queries) - Disabled determination of my location - Deactivated delivery of personalized advertising - Other measures - No, not yet - but I was aware that it is possible - No - I was not aware that this was possible		6, adjustments by: 9
XII) User profile	12.1	In what way do you use search engines?		Please mark the appropriate answer in each case: - By typing in my search query - By submitting my search query by voice - *frequently* - *occasionally* - *rarely* - *never* - *I don’t know*		10
	12.2	In a regular week, for how long do you use the internet approximately?	Scale from 1-7 (days per week)	Please indicate the average number of hours per week: - less than 3 hours per week - 3 to under 6 hours per week - 6 to under 10 hours per week - 10 to under 20 hours per week - 20 to under 30 hours per week - 30 to under 40 hours per week - 40 and more hours per week - I don’t know		4, adjustments by: 9
	12.3	Which of the following activities do you mainly pursue?	- in training or studies - working - unemployed or no longer employed	- employee or public official - self-employed person, freelancer, entrepreneur - student - trainee, apprentice - pupil - housewife/houseman - occasionally employed - not or no longer employed - other		7, adjustments by: 9
	12.4	Which of the following topics play a role in your professional activity?		Please mark all applicable answers: - purchasing, procurement, logistics - finance, controlling - marketing, sales, distribution - IT - digitalization, internet - e-commerce, online trading - online marketing, social media - production, manufacturing - law - none of them	Question for employed internet users. Examine whether people with “SEO-related” professions (e.g., e-commerce) have a different perspective on SEO.	2
	12.5	Which of the following topics belong to your training/studies?		Please mark all applicable answers: - business studies or economics - informatics, business informatics - engineering, electrical engineering - digitalization, internet - e-commerce, online trading - online marketing, social media - law - pedagogy - social sciences - none of them	Question to internet users who are still in training. Check whether people with “SEO-related” topics in training/studies (e.g., e-commerce) have a different perspective on SEO.	2
	12.6	What is your highest educational level?	- None - Certificate of Secondary Education - General Certificate of Secondary Education - university entrance exams - University degree	- Certificate of Secondary Education without completed apprenticeship - Certificate of Secondary Education with completed apprenticeship - General Certificate of Secondary Education - university entrance exams - University degree - None - (Still) without school-leaving certificate (e.g., student) - Other		7, adjustments by: 9

References of the questions: ^1^: Project goals “SEO Effect”, ^2^: Expert interviews ( Schultheiß & Lewandowski, 2021d), ^3^: ( Lewandowski, 2017), ^4^: ( Stark *et al*., 2014), ^5^: ( Schweiger, 2003), ^6^: ( Purcell *et al*., 2012), ^7^: ( Lewandowski & Sünkler, 2013), ^8^: ( Schultheiß & Lewandowski, 2021b) ^9^: Market research company “Fittkau & Maaß”, ^10^: Project staff, *This is a simplified explanation. The fact that a payment to Google is only made after an ad is selected was left unmentioned for the sake of comprehensibility.

SEO: Search engine optimization, PSM: Paid search marketing, SERP: Search engine results page

To give the subjects the opportunity to obtain background information on the survey and to be able to contact the project team, e.g., for feedback purposes, we provided a link to our website at the end of the survey.

The subjects completed 12 sections within the survey as shown in Table 1:

I. Screening
II. Usage behavior
III. Self-assessed search engine literacy
IV. Trust in search engines
V. Query match
VI. Knowledge of search result influences
VII. Knowledge of keyword-related advertisements (i.e., paid search marketing (PSM), ( Li *et al*., 2014))
VIII. Knowledge of SEO
IX. Ability to distinguish ads from organic results
X. Assessments and opinions regarding SEO
XI. Personalization
XII. User profile

The authors in collaboration with F&M have taken care to ensure that the questions are formulated in a way that is understandable for all respondents in the sample. Most of the questions are closed questions. They include rating-scale questions, single and multiple response questions, and questions with marking options for search engine results page (SERP) screenshots. In addition, the survey includes open-ended questions, e.g., “What do you think: Where does Google generate most of its revenue from?” Open-ended questions are particularly suitable for knowledge questions, since in contrast to closed questions, it is not possible to answer a question correctly by chance. A disadvantage of open-ended questions is the required subsequent coding of the answers ( Krosnick & Presser, 2010).

The survey was conducted in the German language. The translated questionnaire is shown in Table 1. The names of the corresponding variables within the data set is included in our research data ( Schultheiß *et al*., 2022) and the original questionnaire in German can be found as part of the research data ( Schultheiß *et al*., 2022).

### Marking tasks

We created eight SERP screenshots for the marking tasks A-D (each task in variants “large screen” and “small screen”). The screenshots are available as part of the research data ( Schultheiß *et al*., 2021).

SERPs A and B were assigned to block I (simple), SERPs C and D to block II (difficult). Two blocks were created to address a variety of SERP elements and to differentiate between basic and complex SERPs. The structure of the two SERPs per block is identical in terms of the elements on the SERP.

Each participant received two tasks, one from block I and one from block II, as shown in Table 2. The SERP for each task was shown two times. First, all ads were to be marked and second, all organic results.

**Table 2.** Marking tasks: queries and elements of SERPs.

Block	Task	Query English (German)	Elements on SERP
block I (simple)	**A**	tax return help (steuererklärung hilfe)	‐ Organic results (10*) ‐ Text ads, top (2*) ‐ Text ads, bottom (2*)
	**B**	legal advice (rechtsberatung)	‐ Organic results (10*) ‐ Text ads, top (2*) ‐ Text ads, bottom (2*)
block II (difficult)	**C**	apple iphone	‐ Organic results (6*) ‐ Text ads, top (2*) ‐ Shopping ads (large screen: 8*, small screen: 2*) ‐ News (large screen: 3*, small screen: 2*) ‐ Knowledge Graph
	**D**	samsung galaxy	‐ Organic results (6*) ‐ Text ads, top (2*) ‐ Shopping ads (large screen: 8*, small screen: 2*) ‐ News (large screen: 3*, small screen: 2*) ‐ Knowledge Graph

SERP: Search engine results page

The screenshots were created using the desktop version of the Chrome browser:

1. User agent: The browser extension User-Agent Switcher for Chrome version 1.1.0 was used to simulate the smartphone (group “small screen”) within the desktop browser (group “large screen”):
  a. Large screen: default
  b. Small screen: Android
2. Window size and page zoom: To create screenshots with high resolution, we used the following settings:
  a. Large screen: Full screen with 400% browser zoom resulted in screenshots with a width of 4436 pixels (px).
  b. Small screen: A browser zoom of 300% resulted in screenshots with a width of 984 px, where the horizontally displayed results (e.g., shopping results) were not cut off/cut in half.
    i. Both zoom settings (400%/300%) were also the highest possible settings for the screenshot tool to capture the entire SERPs.
3. Screenshot: The add-on GoFullPage version 7.1 was used to capture full-page SERP screenshots as PNG files. For each query, the first three SERPs were saved to be able to exchange results during later image processing.
4. Image processing: We used GIMP version 2.10.14 (GIMP development team, 2020) (RRID:SCR_003182) to reduce the SERPs to the elements we wanted to investigate (see Table 2). We also matched the small screen SERPs with the large screen SERPs in terms of results and their positions. Otherwise, different selection behavior in the survey might not have been due to the SERP layout (large vs. small screen), but to partially different results (positions):
  a. Large screen:
    i. The large screen SERPs were reduced to the elements required in the survey, i.e., without “related searches”, “people also ask”.
    ii. Due to the specifications of F&M, the final large screen SERPs were reduced to a width of 800 px.
  b. Small screen:
    i. The results of the small screen SERPs as well as their positions were aligned with the large screen SERPs. Consequently, the large and small screen SERPs for a query only differed in terms of layout, but not in terms of results and their positions.
    ii. Due to the specifications of F&M, the final large screen SERPs were reduced to a width of 360 px.

### Flowchart

**Figure 1.**
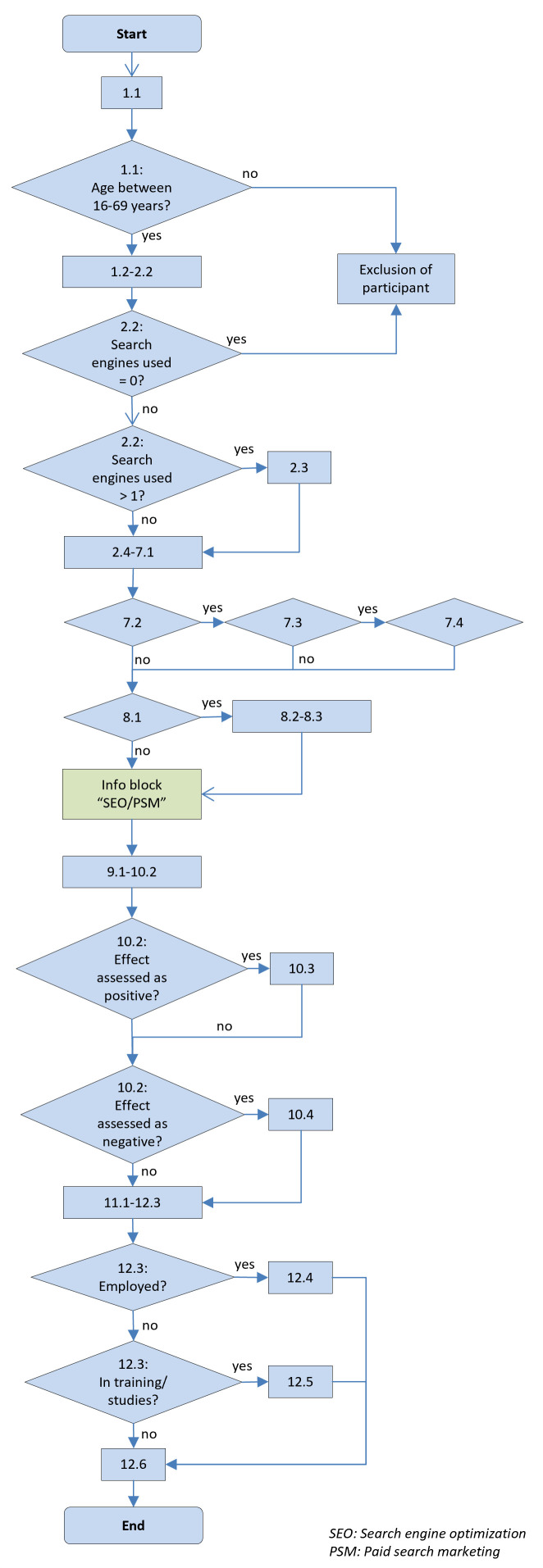
Flowchart of the survey.

Figure 1 shows the flowchart of the online survey.

### Pre-test

Before the survey was conducted, pre-tests were carried out in February 2020 by the members and student assistants of the research group ( *N* = 7) and by the panel provider. This enabled us to test whether problems arose, e.g., regarding comprehensibility, and to eliminate them beforehand.

In the pre-test, problems arose regarding the plausibility of the questionnaire which needed to be fixed before launching the survey. The panel provider checked the survey internally with colleagues to ensure that it was coherent and comprehensible. The duration of the survey was also checked. The maximum duration of 15 minutes as recommended by F&M was met in the pre-tests. Suggestions of the pre-test subjects were also incorporated. These concerned some minor aspects, such as the optical highlighting of relevant parts of a question (e.g., “Are there any search results on this page that can be influenced *by search engine optimization*?”). These recommendations were also implemented. After the pre-test, the *soft launch* started, in which the responses of those subjects who completed the survey first were carefully analyzed. Since the soft launch was successful, the survey could start as planned and the data of the soft launch subjects could also be included in the analysis.

### Ethical approval

Due to the design of the research, we consider the study to be of very low risk for participants. Accordingly, we did not obtain ethical approval. The market research company (F&M), which carried out the survey in cooperation with us, operates according to the principles of the UN Global Compact. This means that F&M operates in a way that fulfils fundamental values regarding human rights, labour, environment, and anti-corruption. Written consent to process their data was obtained from all participants. When registering with online panel provider respondi, participants agreed to the use of their data. For those participants who were minors (16 and 17 years old), parental consent was not required, since “the processing of the personal data of a child shall be lawful where the child is at least 16 years old” (see Article 8 EU GDPR). Data were analysed anonymously. We had no direct contact to the subjects.

### Processing of the data

***Coding and grouping***

Table 3 lists the open-ended questions and the coding specifications. The answers to the knowledge questions were only differentiated into “correct”, “partly correct”, and “incorrect”, since no specifications were made regarding the number of elements (e.g., SEO techniques; question no. 7.3) to be mentioned. The coding of the open-ended questions was done by one coder, which we considered adequate because the coding did not leave any significant room for interpretation.

**Table 3.** Coding of open-ended questions.

No.	Question	Coding
2.2	If you are searching for something online: Which search engine(s) do you usually use? Others, namely… (free input)	- Search engine: e.g., “Baidu“ - Browser: e.g., “Firefox“ - Unsuitable answer: e.g., “Wikipedia”
2.5	Why is [search engine] the search engine you use most often? Please mark up to 5 answers. Other reason, namely… (free input)	- Sustainable/social: e.g., “they plant trees“ - Privacy - Technical advantages: e.g., “easy to use with keywords” - Quality: e.g., “more results than other search engines” - Habit - Against Google: e.g., “I think Google is too powerful” - Pro Google: e.g., “I like that Google pays attention to its users”
6.1	When it comes to the search results displayed on Google: What do you think influences the ranking of search results on Google?	- Payment - Algorithm - Query of the searcher: e.g., “order of terms“ - Tools for website optimization - Traffic/ranking of the website: e.g., “number of clicks“ - User behavior: e.g., “search history“ - User’s Google profile: e.g., “my personal data“ - Topicality/quality/seriousness of the website: e.g., “quality and relevance criteria in terms of content and technology“ - Google’s self-interests - Other: e.g., “No idea. Google gives little information on this“
7.1	What do you think: Where does Google generate most of its revenue from?	- Correct: “ads“ or terms having the same meaning (e.g., advertisement, sponsored results, search engine advertising, SEA, paid search marketing) - Partly correct: correct term (e.g., ads) and at least one incorrect term - Incorrect: clearly incorrect terms (e.g., data sale, donations)
7.4	And how do the paid search results on Google differ from the other results that have not been paid for?	- Correct: “ad label“ or terms having the same meaning (e.g., ad, ad term, label, marking), with or without mentioning the separate position of the ads - Partly correct: correct term (e.g., ad label) and at least one incorrect term - Unclear: only position named as characteristic (e.g., "always the top results") - Incorrect: clearly incorrect terms (e.g., different font)
8.2	Do you know what term is used to describe these measures to improve the ranking in the Google search results list (without payment to Google)?	- Correct: “search engine optimization” or terms having the same meaning (e.g., SEO) - Partly correct: correct term (e.g., SEO) and at least one incorrect term - Incorrect: clearly incorrect terms (e.g., ads, bots)
8.3	And by what means can a website be designed or programmed so that it is ranked higher in the Google search results lists?	- Correct: “keywords” or other correct SEO techniques - Partly correct: correct term (e.g., keywords) and at least one incorrect term; or only “SEO” - Incorrect: clearly incorrect SEO techniques (e.g., payment, ads)
10.3	Which positive effects does search engine optimization have in your opinion?	- Better/more relevant results: e.g., “best result on position 1” - Quicker retrieval: e.g., “you find what you’re looking for faster” - Advantages for the searcher such as individualization, filters: e.g., “the search engine knows me” - Advantages for website operators: e.g., “optimized pages receive more clicks” - Other: e.g., “correction of spelling mistakes”
10.4	Which negative effects does search engine optimization have in your opinion?	- Negative influence on results quality: e.g., “first result not always the best” - (Conscious) influence, manipulation of the results with negative background: e.g., “no objective results” - Displacement of the actually searched, desired, suitable search results: e.g., “commerce and profit comes before truth” - Discrimination against smaller websites/providers: e.g., “distortion of information in favor of solvent website providers” - Other: e.g., “you have to pay attention”

SEO: Search engine optimization, SEA: Search engine advertising

Table 4 shows how the topics from professional activity, training, and studies have been grouped in terms of SEO affinity (low, average, high). To group the topics, we examined module handbooks of the studies for intersections with the SEO topic. In the case of training and professional activity, e.g., pedagogy, we examined corresponding studies, e.g., educational science.

**Table 4.** Affinity to SEO topics (grouping).

Response options	Affinity to SEO
*Question no. 12.4: Which of the following topics play a role in your professional activity?*	
purchasing, procurement, logistics	low
finance, controlling	low
production, manufacturing	low
law	low
marketing, sales, distribution	average
IT	average
digitalization, internet	high
e-commerce, online trading	high
online marketing, social media	high
*Question no. 12.5: Which of the following topics belong to your training/studies?*	
business studies or economics	low
engineering, electrical engineering	low
law	low
pedagogy	low
social sciences	low
informatics, business informatics	average
digitalization, internet	high
e-commerce, online trading	high
online marketing, social media	high

SEO: Search engine optimization, IT: Information technology

***Success rates for marking tasks***

Table 5 shows the search results to be marked on the SERPs according to the task, device, and area (SEO or PSM).

**Table 5.** Marking tasks: results to be marked.

Task	Device	Area	Results to be marked
A	Large screen & small screen	SEO	- Organic results (10*)
A	Large screen & small screen	PSM	- Text ads, top of SERP (2*) - Text ads, bottom of SERP (2*)
B	Large screen & small screen	SEO	- Organic results (10*)
B	Large screen & small screen	PSM	- Text ads, top of SERP (2*) - Text ads, bottom of SERP (2*)
C	Large screen	SEO	- Organic results (6*) - News (3*)
C	Large screen	PSM	- Text ads, top of SERP (2*) - Shopping ads (8*)
C	Small screen	SEO	- Organic results (6*) - News (2*)
C	Small screen	PSM	- Text ads, top of SERP (2*) - Shopping ads (2*)
D	Large screen	SEO	- Organic results (6*) - News (3*)
D	Large screen	PSM	- Text ads, top of SERP (2*) - Shopping ads (8*)
D	Small screen	SEO	- Organic results (6*) - News (2*)
D	Small screen	PSM	- Text ads, top of SERP (2*) - Shopping ads (2*)

SEO: Search engine optimization, PSM: Paid search marketing

Based on the marked elements, a success rate was calculated for each participant per task (A-D), device (large, small), and area (SEO, PSM). This rate accounts for correctly marked (true positive) and incorrectly marked (false positive) results using the formula $\frac{n\;\text{true}-n\;\text{false}}{n\;\text{to}\;\mathrm{be}\;\text{marked}}$ .

Two examples follow, the first for achieving a positive success rate for task A, large screen, SEO results. In this case, 10 organic results are to be marked, of which the subject marks 8 results (8 true). In addition, the subject incorrectly marks 2 ads (2 false). This results in a success rate of 0.6. Negative success rates are also possible, if a subject makes more incorrect than correct markings, exemplified by task B, small screen, PSM results. In this case, a total of 4 text ads are to be marked. If a subject identifies all 4 text ads (true), but additionally marks 6 organic results (false), the subject achieves a success rate of -0.5.

For the calculation of the success rates and the corresponding variables of the data set, see Appendix 1: Calculation of success rates.

## Data availability

### Underlying data

OSF: SEO-Effekt/Online survey. https://doi.org/10.17605/OSF.IO/PG82E ( Schultheiß *et al*., 2022)

This project contains the following underlying data:

- Survey data.xlsx (full data set of representative online survey)

### Extended data

OSF: SEO-Effekt/Online survey. https://doi.org/10.17605/OSF.IO/PG82E ( Schultheiß *et al*., 2022)

This project contains the following extended data:

- SERPs.zip (screenshots of SERPs for marking tasks)
- variables English (names and descriptions of all variables; English)
- variables German (names and descriptions of all variables; German)
- Working Paper_online survey.pdf (Working paper with information on background, methods, and results of the survey)

Data are available under the terms of the Creative Commons Attribution 4.0 International license (CC-BY 4.0).

## Other required information

## Publications that use the data

• ( Lewandowski & Schultheiß, 2022)
• ( Schultheiß & Lewandowski, 2021a)
• ( Schultheiß & Lewandowski, 2021c)

## Appendix 1: Calculation of success rates

**Table:**

Step 1: Calculation of correct and incorrect markings		
Calculation *(addition of variables and dividing by 2, as each marked result is recorded with "2".)*	Description *(Task, SEO or PSM, device, results type)*	New Variable *(from the calculation shown in the left column, a new variable is created for step 2 explained below)*
(ad201+ad202+ad213+ad214)/2 *Example: (2+0+2+2)/2=3 ➔ 3 text ads were marked for task A on large screen when ads were to be marked (results type „PSM“)*	Task A, PSM, large screen, text ads	**ad2t01** *➔ Thus, „ **ad2t01**” indicates the number of text ads that have been marked in “task A, PSM results, large screen” (in our example, 3 text ads)*
(ad203+ad204+ad205+ad206+ad207+ad208+ad209+ad210+ad211+ad212)/2	Task A, PSM, large screen, organic results	**ad2o01** *➔ Number of organic results that have been marked in “task A, PSM results, large screen”*
(ad401+ad402+ad413+ad414)/2	Task A, SEO, large screen, text ads	ad4t01
(ad403+ad404+ad405+ad406+ad407+ad408+ad409+ad410+ad411+ad412)/2	Task A, SEO, large screen, organic results	ad4o01
(bd201+bd202+bd213+bd214)/2	Task B, PSM, large screen, text ads	bd2t01
(bd203+bd204+bd205+bd206+bd207+bd208+bd209+bd210+bd211+bd212)/2	Task B, PSM, large screen, organic results	bd2o01
(bd401+bd402+bd413+bd414)/2	Task B, SEO, large screen, text ads	bd4t01
(bd403+bd404+bd405+bd406+bd407+bd408+bd409+bd410+bd411+bd412)/2	Task B, SEO, large screen, organic results	bd4o01
(am201+am202+am213+am214)/2	Task A, PSM, small screen, text ads	am2t01
(am203+am204+am205+am206+am207+am208+am209+am210+am211+am212)/2	Task A, PSM, small screen, organic results	am2o01
(am401+am402+am413+am414)/2	Task A, SEO, small screen, text ads	am4t01
(am403+am404+am405+am406+am407+am408+am409+am410+am411+am412)/2	Task A, SEO, small screen, organic results	am4o01
(bm201+bm202+bm213+bm214)/2	Task B, PSM, small screen, text ads	bm2t01
(bm203+bm204+bm205+bm206+bm207+bm208+bm209+bm210+bm211+bm212)/2	Task B, PSM, small screen, organic results	bm2o01
(bm401+bm402+bm413+bm414)/2	Task B, SEO, small screen, text ads	bm4t01
(bm403+bm404+bm405+bm406+bm407+bm408+bm409+bm410+bm411+bm412)/2	Task B, SEO, small screen, organic results	bm4o01
(cd201+cd202)/2	Task C, PSM, large screen, text ads	cd2t01
(cd203+cd204+cd205)/2	Task C, PSM, large screen, news results	cd2n01
(cd206+cd207+cd208+cd209+cd210+cd211)/2	Task C, PSM, large screen, organic results	cd2o01
(cd212+cd213+cd214+cd215+cd216+cd217+cd218+cd219)/2	Task C, PSM, large screen, shopping ads	cd2s01
(cd220)/2	Task C, PSM, large screen, knowledge graph	cd2w01
(cd401+cd402)/2	Task C, SEO, large screen, text ads	cd4t01
(cd403+cd404+cd405)/2	Task C, SEO, large screen, news results	cd4n01
(cd406+cd407+cd408+cd409+cd410+cd411)/2	Task C, SEO, large screen, organic results	cd4o01
(cd412+cd413+cd414+cd415+cd416+cd417+cd418+cd419)/2	Task C, SEO, large screen, shopping ads	cd4s01
(cd420)/2	Task C, SEO, large screen, knowledge graph	cd4w01
(dd201+dd202)/2	Task D, PSM, large screen, text ads	dd2t01
(dd203+dd204+dd205)/2	Task D, PSM, large screen, news results	dd2n01
(dd206+dd207+dd208+dd209+dd210+dd211)/2	Task D, PSM, large screen, organic results	dd2o01
(dd212+dd213+dd214+dd215+dd216+dd217+dd218+dd219)/2	Task D, PSM, large screen, shopping ads	dd2s01
(dd220)/2	Task D, PSM, large screen, knowledge graph	dd2w01
(dd401+dd402)/2	Task D, SEO, large screen, text ads	dd4t01
(dd403+dd404+dd405)/2	Task D, SEO, large screen, news results	dd4n01
(dd406+dd407+dd408+dd409+dd410+dd411)/2	Task D, SEO, large screen, organic results	dd4o01
(dd412+dd413+dd414+dd415+dd416+dd417+dd418+dd419)/2	Task D, SEO, large screen, shopping ads	dd4s01
(dd420)/2	Task D, SEO, large screen, knowledge graph	dd4w01
(cm201+cm202)/2	Task C, PSM, small screen, shopping ads	cm2s01
(cm203+cm204)/2	Task C, PSM, small screen, text ads	cm2t01
(cm205+cm206+cm211+cm212+cm213+cm214)/2	Task C, PSM, small screen, organic results	cm2o01
(cm208+cm209)/2	Task C, PSM, small screen, news results	cm2n01
(cm210)/2	Task C, PSM, small screen, knowledge graph	cm2w01
(cm401+cm402)/2	Task C, SEO, small screen, shopping ads	cm4s01
(cm403+cm404)/2	Task C, SEO, small screen, text ads	cm4t01
(cm405+cm406+cm411+cm412+cm413+cm414)/2	Task C, SEO, small screen, organic results	cm4o01
(cm408+cm409)/2	Task C, SEO, small screen, news results	cm4n01
(cm410)/2	Task C SEO, small screen, knowledge graph	cm4w01
(dm201+dm202)/2	Task D PSM, small screen, shopping ads	dm2s01
(dm203+dm204)/2	Task D, PSM, small screen, text ads	dm2t01
(dm205+dm206+dm211+dm212+dm213+dm214)/2	Task D, PSM, small screen, organic results	dm2o01
(dm208+dm209)/2	Task D, PSM, small screen, news results	dm2n01
(dm210)/2	Task D, PSM, small screen, knowledge graph	dm2w01
(dm401+dm402)/2	Task D, SEO, small screen, shopping ads	dm4s01
(dm403+dm404)/2	Task D, SEO, small screen, text ads	dm4t01
(dm405+dm406+dm411+dm412+dm413+dm414)/2	Task D, SEO, small screen, organic results	dm4o01
(dm408+dm409)/2	Task D, SEO, small screen, news results	dm4n01
(dm410)/2	Task D, SEO, small screen, knowledge graph	dm4w01

**Table:**

Step 2: Calculation of success rates based on variables of step 1		
Calculation *(divided by number of results to be marked)*	Description	New Variable *(for success rates)*
( **ad2t01** - **ad2o01**)/4 *(number of marked ads – number of marked organic results)/number of ads to be marked on SERP)*	Success rate PSM, task A, large screen	ad2sp *Thus, “ad2sp” indicates the success rate for “task A, large screen, PSM results”*
(ad4o01 - ad4t01)/10	Success rate SEO, task A, large screen	ad4so
(bd2t01 - bd2o01)/4	Success rate PSM, task B, large screen	bd2sp
(bd4o01 - bd4t01)/10	Success rate SEO, task B, large screen	bd4so
(am2t01 - am2o01)/4	Success rate PSM, task A, small screen	am2sp
(am4o01 - am4t01)/10	Success rate SEO, task A, small screen	am4so
(bm2t01 - bm2o01)/4	Success rate PSM, task B, small screen	bm2sp
(bm4o01 - bm4t01)/10	Success rate SEO, task B, small screen	bm4so
((cd2t01 + cd2s01) -(cd2n01 + cd2o01 + cd2w01))/10	Success rate PSM, task C, large screen	cd2sp
((cd4o01 + cd4n01) -(cd4t01 + cd4s01 + cd4w01))/9	Success rate SEO, task C, large screen	cd4so
((dd2t01 + dd2s01) -(dd2n01 + dd2o01 + dd2w01))/10	Success rate PSM, task D, large screen	dd2sp
((dd4o01 + dd4n01) -(dd4t01 + dd4s01 + dd4w01))/9	Success rate SEO, task D, large screen	dd4so
((cm2s01 + cm2t01) -(cm2o01 + cm2n01 + cm2w01))/4	Success rate PSM, task C, small screen	cm2sp
((cm4o01 + cm4n01) -(cm4s01 + cm4t01 + cm4w01))/8	Success rate SEO, task C, small screen	cm4so
((dm2s01 + dm2t01) -(dm2o01 + dm2n01 + dm2w01))/4	Success rate PSM, task D, small screen	dm2sp
((dm4o01 + dm4n01) -(dm4s01 + dm4t01 + dm4w01))/8	Success rate SEO, task D, small screen	dm4so
