# Supplementary material for: Data set of a representative online survey on search engines with a focus on search engine optimization (SEO): a cross-sectional study
Source: F1000Res. 2022 Sep 12;11:376. Originally published 2022 Mar 31. [Version 2] doi: 10.12688/f1000research.109662.2 (PMC9551386; doi:10.12688/f1000research.109662.2)
Supplement: Peer review history

# Peer review: Reviewer response for version 2

**DOI:** 10.5256/f1000research.137147.r150168

**Published:** 2022-10-10

**Authors:** Melius Weideman

**Affiliations:** 1 Cape Peninsula University of Technology, Cape Town, South Africa

Ready to be indexed.

Are sufficient details of methods and materials provided to allow replication by others?

Yes

Is the rationale for creating the dataset(s) clearly described?

Yes

Are the datasets clearly presented in a useable and accessible format?

Yes

Are the protocols appropriate and is the work technically sound?

Partly

Reviewer Expertise:

SEO, information retrieval

I confirm that I have read this submission and believe that I have an appropriate level of expertise to confirm that it is of an acceptable scientific standard.

# Response

**Published:** 2022-10-10

**Authors:** Sebastian Schultheiß

**Affiliations:** Hamburg University of Applied Sciences, Germany

Dear Melius Weideman,

 ​​​

 Thank you very much for taking the time to review the second version of our Data Note. We are pleased about the positive feedback.

 Best regards from Hamburg,

 Sebastian Schultheiß and Dirk Lewandowski

# Peer review: Reviewer response for version 1

**DOI:** 10.5256/f1000research.121194.r144671

**Published:** 2022-08-04

**Authors:** Lluís Codina

**Affiliations:** 1 Universitat Pompeu Fabra, Barcelona, Spain

This paper presents in detail a complete methodology to carry out online surveys among German search engine users. In addition to the explanation of the methodology, and its various steps, it includes theoretical foundations. The way to proceed step by step and the precautions used for the validity of the work are explained in detail. The article also presents the complete tables and schemes used for the survey, which provides a high value to this work.

 The rationale presented for the creation of the data set is clearly indicated. The research it supports is necessary and timely as it fills in clearly identified research gaps. In addition, the data set is rich and conceptually very powerful with clear possibilities to support ambitious investigations.

 On the other hand, the protocols used are described exhaustively and with sufficient technical and conceptual details. The article points out that an ethical approval was not required, but they convincingly explain the reasons for it, and the guarantees adopted by both the researchers and the contracted company.

 For the above reasons, the dataset is traceable, which is why other researchers can analyze and replicate the research.

Are sufficient details of methods and materials provided to allow replication by others?

Yes

Is the rationale for creating the dataset(s) clearly described?

Yes

Are the datasets clearly presented in a useable and accessible format?

Yes

Are the protocols appropriate and is the work technically sound?

Yes

Reviewer Expertise:

Search Engine Optimization, Digital News Media

I confirm that I have read this submission and believe that I have an appropriate level of expertise to confirm that it is of an acceptable scientific standard.

# Response

**Published:** 2022-09-05

**Authors:** Sebastian Schultheiß

**Affiliations:** Hamburg University of Applied Sciences, Germany

Dear Lluís Codina,

 Thank you very much for taking the time to review our data note. We are pleased about the positive review.

 Best regards,

 Sebastian Schultheiß and Dirk Lewandowski

# Peer review: Reviewer response for version 1

**DOI:** 10.5256/f1000research.121194.r129422

**Published:** 2022-07-04

**Authors:** Melius Weideman

**Affiliations:** 1 Cape Peninsula University of Technology, Cape Town, South Africa

On a serious note: 

1. The authors claim that "we did not obtain ethical approval", but at the same time participants were paid for their participation, albeit a small amount. The moment there is any payment involved, ethical clearance is a must. Even if no payment was involved, ALL university research must go through an ethical approval process.
2. The amount of self-citation in this article is extreme. 8/18 of the references (that is about 44%) are the authors themselves! This is unacceptable in the academic world.

These two issues are serious enough to put a question mark over this research.

 Furthermore there are a few minor issues with this article:

1. No results are given in the abstract. That is standard practice, and some indication of the results must be given in an abstract.
2. Some mindless repetition is evident. The phrases "with N=2.012" and "from the age of 16 to 69 years" appear numerous times, without adding value.
3. Double dots appear in the "Materials and methods" section.
4. It is not clear what Table 1 is supposed to present.
5. The term "open questions" is often used. This should probably be "open-ended questions".
6. "conducted in German language" should be "conducted in the German language".
7. The reference to Table 1 on page 4 is wrong. There is no translated questionnaire in Table 1.
8. There are some errors in the flow-chart of Figure 1. Almost halfway from the top, the row with the diamonds named 7.2, 7.3 and 7.4 and the next row 8.1, are incomplete. What is the condition tested for? Was the answer 7.2 or 7.3, etc?
9. On p8, under "Pre-test", "problems arise" must be "problems arose".
10. Just below Table 5, The formula is written in strange and different fonts.
11. In the 2nd paragraph after Table 5, how is the calculation done to arrive at 0.6?

Are sufficient details of methods and materials provided to allow replication by others?

Yes

Is the rationale for creating the dataset(s) clearly described?

Yes

Are the datasets clearly presented in a useable and accessible format?

Yes

Are the protocols appropriate and is the work technically sound?

Partly

Reviewer Expertise:

SEO, information retrieval

I confirm that I have read this submission and believe that I have an appropriate level of expertise to confirm that it is of an acceptable scientific standard, however I have significant reservations, as outlined above.

# Response

**Published:** 2022-09-05

**Authors:** Sebastian Schultheiß

**Affiliations:** Hamburg University of Applied Sciences, Germany

Dear Melius Weideman,

 Thank you very much for taking the time to review our data note. We are happy to address your comments.

1. "The authors claim that "we did not obtain ethical approval", but at the same time participants were paid for their participation, albeit a small amount. The moment there is any payment involved, ethical clearance is a must. Even if no payment was involved, ALL university research must go through an ethical approval process."
  - We agree that, although we did not consider it necessary, it would have been better to obtain ethical approval for our study. For future studies, we will therefore obtain ethical approval for all studies without exception.
2. "The amount of self-citation in this article is extreme. 8/18 of the references (that is about 44%) are the authors themselves! This is unacceptable in the academic world."
  - Thank you for the criticism, which we would agree with in the case of regular research articles. However, since our article is a data note, we have a different view on self-citation, which we are glad to clarify.
    As specified by F1000research, the focus of data notes is a comprehensive description of the data, but not an extensive introduction or extensive literature sections. On the one hand, this justifies the low number of cited references. On the other hand, it also explains the high self-citation rate since the online survey methodology described in the data note is directly related to our preliminary work.
3. "No results are given in the abstract. That is standard practice, and some indication of the results must be given in an abstract."
  - Since the article is a data note, it does not include any results, which is why the abstract does not include any results either. This is in line with the F1000reserarch guidelines for data notes: “Data Notes are brief descriptions of datasets that promote the potential reuse of research data and include details of why and how the data were created; they do not include any analyses or conclusions.” (see https://f1000research.com/for-authors/article-guidelines)
4. "It is not clear what Table 1 is supposed to present."
  - Table 1 shows the questionnaire. It contains the questions the subjects were asked, the response options, and the sources of the respective questions. Could you please elaborate on what is unclear about the table from your perspective?
5. "The reference to Table 1 on page 4 is wrong. There is no translated questionnaire in Table 1."
  - Since the study was conducted in German, the English version of the questionnaire shown in Table 1 *is* the translated questionnaire. Thus, we have not made any changes.
6. "There are some errors in the flow-chart of Figure 1. Almost halfway from the top, the row with the diamonds named 7.2, 7.3 and 7.4 and the next row 8.1, are incomplete. What is the condition tested for? Was the answer 7.2 or 7.3, etc?"
  - After the open-ended question 7.1, all subjects received the closed question 7.2:
    - If the answer to this question was "yes," the next question was 7.3, which was also a closed question. If the answer to this question was again "yes," the next question was open-ended question 7.4. Then, question 8.1 followed for all participants.
    - If the answer to the closed question 7.2 was “no,” question 8.1 followed immediately, without questions 7.3 and 7.4 being asked.
    - However, we are unsure if we have understood you correctly. Could you please explain where exactly you see errors in the flowchart?
7. "Just below Table 5, The formula is written in strange and different fonts."
  - As authors, we unfortunately cannot influence the formatting of the formula. @F1000research: Please match the font of the formula to the font of the rest of the text, if possible.
8. "In the 2nd paragraph after Table 5, how is the calculation done to arrive at 0.6?"
  - The example is calculated as follows: 8 “true” (these are the correctly marked 8 organic results) - 2 “false” (these are the incorrectly marked 2 ads) / 10 “to be marked” (these are the 10 organic results that should have been marked) = success rate of 0.6.

We have implemented the following comments as proposed:

1. Some mindless repetition is evident. The phrases "with N=2.012" and "from the age of 16 to 69 years" appear numerous times, without adding value.
2. Double dots appear in the "Materials and methods" section.
3. The term "open questions" is often used. This should probably be "open-ended questions".
4. "conducted in German language" should be "conducted in the German language".
5. On p8, under "Pre-test", "problems arise" must be "problems arose".

Best regards,

 Sebastian Schultheiß and Dirk Lewandowski
